# Evidence for an increase in the intensity of inter‐seasonal influenza, Queensland, Australia, 2009‐2019

**DOI:** 10.1111/irv.12828

**Published:** 2020-12-24

**Authors:** Elenor J. Kerr, Jonathan Malo, Kaitlyn Vette, Graeme R. Nimmo, Stephen B. Lambert

**Affiliations:** ^1^ National Centre for Epidemiology and Population Health Australian National University Canberra ACT Australia; ^2^ Communicable Diseases Branch Queensland Health Brisbane Qld Australia; ^3^ National Centre for Immunisation Research and Surveillance Sydney NSW Australia; ^4^ Pathology Queensland Queensland Health Brisbane Qld Australia

**Keywords:** Australia, epidemiology, human, influenza, inter‐seasonality, seasons, surveillance

## Abstract

**Background:**

Inter‐seasonal influenza cases have been increasing in Australia. Studies of influenza seasonality typically focus on seasonal transmission in temperate regions, leaving our understanding of inter‐seasonal epidemiology limited. We aimed to improve understanding of influenza epidemiology during inter‐seasonal periods across climate zones, and explored influenza intensity and strain dominance patterns over time.

**Methods:**

Queensland state‐wide laboratory‐confirmed influenza notifications and public laboratory influenza test data from 2009‐2019 were described by demographics, time period, region and strain type. We compared influenza intensity over time using the WHO Average Curve method to provide thresholds for seasonal and inter‐seasonal periods.

**Results:**

Among the 243 830 influenza notifications and 490 772 laboratory tests reported in Queensland between 2009 and 2019, 15% of notifications and 40% of tests occurred during inter‐seasonal periods, with 6.3% of inter‐seasonal tests positive. Inter‐seasonal notifications and tests substantially increased over time and increases in weekly proportions positive and intensity classifications suggested gradual increases in virus activity. Tropical inter‐seasonal activity was higher with periods of marked increase. Influenza A was dominant, although influenza B represented up to 72% and 42% of notifications during some seasonal and inter‐seasonal periods, respectively.

**Conclusions:**

Using notification and testing data, we have demonstrated a gradual increase in inter‐seasonal influenza over time. Our findings suggest this increase results from an interplay between testing, activity and intensity, and strain circulation. Seasonal intensity and strain circulation appeared to modify subsequent period intensity. Routine year‐round surveillance data would provide a better understanding of influenza epidemiology during this infrequently studied inter‐seasonal time period.

## 1 BACKGROUND

In temperate regions human influenza typically has a strong seasonal cycle where influenza activity increases between late autumn and early spring, before returning to baseline activity during warmer months.^1^, ^2^ Influenza cases during the summer inter‐seasonal period are often considered to be sporadic imported cases unlikely to cause substantial ongoing transmission.^3^, ^4^, ^5^ However, recent evidence suggests that inter‐seasonal transmission patterns are more complex than previously thought.^3^ In tropical and sub‐tropical regions, inter‐seasonal influenza patterns are variable with some areas experiencing increased activity during rainy seasons, and others not experiencing a well‐defined season.^2^, ^6^ Regardless of the climate zone, outbreaks of influenza may also occur outside of the typical season as evidenced by the large and widespread influenza outbreaks across Australia during the 2018/19 inter‐seasonal period.^7^

Changes to influenza reporting, testing capacity and testing behaviour likely play a role in increased recognition of influenza infections during inter‐seasonal periods, both globally and in Australia.^1^ Nonetheless, higher than average inter‐seasonal influenza notifications, beyond what could be accounted for by increased testing and reporting, have been observed previously in Australia during the 2010/11 and 2018/19 inter‐seasonal periods.^7^, ^8^

Recent Australian analyses provide insights into Australian inter‐seasonal activity, transmission and testing practices, yet previous studies of influenza seasonality typically focus on transmission within seasonal periods in temperate regions.^1^, ^3^, ^7^, ^9^ Our understanding of the epidemiology of inter‐seasonal periods across different climatic regions over time therefore remains limited.

Here we use routinely collected surveillance data from 2009 to 2019 to summarize the epidemiology of influenza during inter‐seasonal periods in Queensland over a 10‐year period and explore influenza intensity and strain dominance patterns over time. We focus our analyses on the Australian state of Queensland, which by spanning temperate, sub‐tropical and tropical climates provides insights into patterns of seasonality across different climatic regions.

## 2 METHODS

### 2.1 Study setting and data sources

Queensland is a state with a population of approximately 5 million people located in Australia's northeast. It has a varied climate including temperate, sub‐tropical and tropical zones. Queensland has well‐established influenza surveillance systems which monitor activity using laboratory‐confirmed influenza notifications, public laboratory influenza testing data and hospital admissions for influenza to Queensland public hospitals. Laboratory‐confirmed influenza is a nationally notifiable condition in Australia and has been notifiable on pathological diagnosis in Queensland since 2001. Confirmed cases are notified on laboratory definitive evidence; virus isolation by culture, detection by nucleic acid testing, or antigen detection in appropriate respiratory specimens or seroconversion or a fourfold or greater rise in an antibody titre to influenza virus. Notification is also made on the detection of a single high titre of IgA antibody.

Notifications of laboratory‐confirmed influenza from both public and private laboratories are recorded in Queensland's Notifiable Condition System (NoCS). The Queensland public laboratory information system (AUSLAB), holds laboratory test request and result records of all public hospital inpatients and outpatients, as well as testing records for community clinics and prisons in Queensland. Routinely collected data from the beginning of the 2009 season (01 May) to the end of the 2019 season (03 November) were used for this analysis. Laboratory‐confirmed influenza notifications and data for all individuals tested for influenza using PCR were extracted from NoCS and AUSLAB, respectively. As serology was infrequent during this study period, representing only 2.45% of total testing and 1.90% of positive tests, only notifications and testing data from PCR results, including GeneXpert data, were used.

### 2.2 Analysis

Notification and testing data were analysed as both overall period (seasonal/inter‐seasonal), individual season/inter‐seasonal period, and weekly totals. Weeks were defined using International Organisation for Standardisation (ISO) 8601 standard weeks. Consistent with historical Queensland influenza activity, seasonal and inter‐seasonal periods were fixed; seasons: weeks 22‐44, inter‐seasonal periods: weeks 45‐21.

### 2.3 Inter‐seasonal epidemiology

We examined the distribution of both inter‐seasonal and seasonal influenza periods by age group (<5, 5‐<10, 10‐<20, 20‐<30, 30‐<40, 40‐<50, 50‐<60, 60‐<65, ≥65 years), sex, region and influenza strain type (Influenza A: H1, H3, untyped; Influenza B: Yamagata, Victoria, untyped). Geographical regions (Southern, Central, Tropical) align to those used for state‐level influenza surveillance and Bureau of Meteorology climate zones. A ratio of seasonal:inter‐seasonal influenza notifications, tests, and proportion of tests positive were calculated. The 2009 pandemic season was excluded from ratio calculations due to changes in laboratory testing practices and extreme counts during the H1N1pdm09 pandemic period.^10^

### 2.4 Intensity and strain dominance

To characterize and compare influenza intensity over time, thresholds were set for seasons and inter‐seasonal periods separately using the World Health Organization (WHO) average curve method.^11^ Historic data from 31 May 2010 (beginning of the 2010 season) to 03 November 2019 were aligned on the median week of peak activity and assigned thresholds of “no activity” (below annual median value), “low” (between the annual median value and the upper 40% confidence interval (CI) of the mean peak value of the average curve), “moderate” (between the upper limit of the 40% CI and 90% CI of the mean peak value of the average curve), “high” (between the upper limit of the 90% and 97.5% CI of the mean peak value of the average curve), and “extraordinary” (above the upper limit of the 97.5% CI of the mean peak value of the average curve). Due to large variance in peak values, CIs were calculated using a geometric mean.

Data from the 2010‐2019 seasons were used to calculate the seasonal thresholds and data from the 2010/11‐2018/19 inter‐seasonal periods were used to calculate the inter‐seasonal thresholds with 53rd weeks excluded from analysis. Data from the 2009 season and 2009/10 inter‐seasonal period were excluded. Thresholds used a weekly composite measurement, the product of laboratory‐confirmed notification rates (per 1000 population) and proportion of laboratory tests positive for influenza, and were smoothed using a 3‐week moving average to reduce short‐term fluctuations in values. Such composite influenza measurements are considered a better proxy indicator of influenza incidence than either notification or laboratory data alone as they improve representativeness and account for testing practices and behaviours.^12^, ^13^ Strain dominance was defined as the largest proportion of strain type among all notifications during that period. Influenza B co‐circulation was defined as where influenza B accounted for ≥20% of all notifications for the period.

### 2.5 Ethics statement

This study was approved by the ANU Human Research Ethics Committee (Protocol 2019/442) and approval for access to Queensland Health information was granted under section 284 of the Queensland *Public Health Act 2005*. All data were de‐identified.

## 3 RESULTS

A total of 243 830 influenza notifications and 490 772 laboratory tests were reported in Queensland. Among which, 190 332 (78%) notifications occurred during seasonal periods (excluding the 2009 season), 17 147 (7%) notifications occurred during the 2009 pandemic season, and 36 351 (15%) notifications occurred during inter‐seasonal periods (Table 1).

**Table 1:** Inter‐seasonal laboratory‐confirmed influenza notifications, influenza testing and proportion positive inter‐seasonal to seasonal ratio, Queensland 2009/10 to 2018/19

Characteristic	Notifications				Tests			Proportion positive (%)		
	IS	S^a^	Ratio^b^	IS		S	Ratio	IS	S	Ratio
Total	36 351	190 332	0.19	198 137		267 071	0.74	06.3	18.2	0.35
Age group (y)										
<5	3066	21 891	0.14	68 144		73 084	0.93	02.3	11.8	0.20
5‐<10	2825	23 339	0.12	12 468		18 004	0.69	07.0	24.9	0.28
10‐<20	3834	28 699	0.13	9394		13 746	0.68	10.8	30.1	0.36
20‐<30	3821	20 366	0.19	10 529		16 113	0.65	11.6	27.3	0.43
30‐<40	4343	22 990	0.19	11 048		16 448	0.67	10.6	24.7	0.43
40‐<50	4176	18 964	0.22	12 214		17 245	0.71	09.4	19.8	0.48
50‐<60	4557	16 616	0.27	16 365		23 109	0.71	08.7	17.7	0.49
60‐<65	2187	7866	0.28	9940		13 819	0.72	07.8	17.4	0.45
≥65	7242	29 601	0.24	48 035		75 503	0.64	06.9	17.3	0.40
Sex										
Female	19 641	102 662	0.19	95 148		133 092	0.71	06.8	19.0	0.36
Male	16 710	87 670	0.19	102 989		133 092	0.77	05.9	17.4	0.34
Region										
Tropical	9637	19 912	0.48	39 649		42 351	0.94	11.4	18.2	0.63
Central	12 866	75 518	0.17	70 287		101 176	0.70	05.3	18.1	0.29
Southern	13 848	94 902	0.15	82 505		116 304	0.71	04.7	18.2	0.26
Strain type										
Influenza A (all)	29 608	134 972	0.22							
A H1	3288	7268	0.45							
A H3	3915	10 380	0.38							
Untyped	22 405	117 324	0.19							
Influenza B (all)	6740	55 354	0.12							
Yamagata	205	300	0.68							
Victoria	167	642	0.26							
Untyped	6368	54 412	0.12							

Abbreviations: IS, Inter‐seasonal period (ISO week 45‐21); S, Seasonal period (ISO week 22‐44).

^a^ Excludes the 2009 pandemic season.

^b^ Ratio of inter‐seasonal to seasonal values.

Among laboratory tests, 267 071 (54.4%) were performed during seasonal periods, 25 564 (5.2%) during the 2009 pandemic season, and 198 137 (40.4%) during inter‐seasonal periods. Inter‐seasonal notifications increased from 71 (2009/10) to 15 529 (2018/19) over the study period and tests increased from 9240 to 41 430 (Table 2). Overall 6.3% of tests during inter‐seasonal periods between 2009/10 to 2018/19 were positive (12 549/198 137). The inter‐seasonal period proportion positive ranged between 0.5% (2009/10) and 12.6% (2018/19). There was no clear overall increasing trend across inter‐seasonal periods over time.

**Table 2:** Inter‐seasonal laboratory‐confirmed influenza notifications, testing and proportion positive by period, Queensland 2009/10 to 2018/19

Inter‐seasonal period^a^	Notifications		Tests		Proportion positive^b^ (%)
	N	Weekly median (IQR)	N	Weekly median (IQR)	
2009/10	71	02 (01‐03)	9240	299 (229‐390)	0.5
2010/11	1805	67 (46‐78)	13 174	460 (388‐520)	6.7
2011/12	647	18 (13‐27)	9902	331 (302‐369)	2.2
2012/13	796	23 (15‐40)	11 850	424 (346‐475)	1.9
2013/14	1975	70 (55‐84)	13 459	456 (392‐545)	5.0
2014/15	2138	73 (60‐92)	17 482	562 (521‐693)	4.1
2015/16	3091	84.5 (49‐167)	26 316	893.5 (753‐983)	4.5
2016/17	4750	162 (144‐185)	25 930	916 (836‐948)	6.1
2017/18	5549	196 (137‐246)	29 444	1060 (918‐1109)	6.2
2018/19	15 529	508 (444‐569)	41 340	1322 (1241‐1643)	12.6
Total	36 351		198 137		6.3

Abbreviation: IQR, Interquartile range.

^a^ ISO week 45‐21.

^b^ Proportion of public laboratory influenza tests influenza positive.

GeneXpert testing (103 867 tests, 21.1%) increased from 751 tests in 2015 to 64 285 in 2019. Until 2018 GeneXpert was primarily run in parallel to other PCR testing. Between 2017 and 2019, 11 135 notifications (2.3%) were based on GeneXpert positivity alone.

### 3.1 Inter‐seasonal epidemiology

Increases in notifications and influenza tests over the study period were most evident among the ≥65‐year age group; notifications and tests increased each inter‐seasonal period on average by 61% and 59%, respectively. Thirty‐four per cent of all tests during inter‐seasonal periods were for children younger than 5‐years of age and testing in this age group was high across all inter‐seasonal periods, ranging between 5410 tests (2009/10) and 9539 (2015/16). However, the proportion positive for children younger than 5 years of age was the lowest of all age groups (1592/68 144, 2.3%) and increases in notifications over the study period were consistent across all age groups, apart from the ≥65‐year age group.

**Figure 1:**
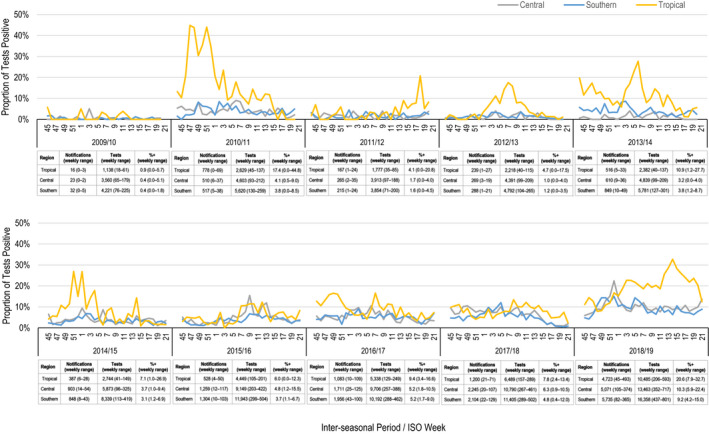
Inter‐seasonal proportion of influenza test positive by region and International Organisation for Standardisation (ISO) weeks of laboratory test, Queensland November 2009/10 to 2018/19

The Southern and Central regions accounted for 38.1% and 35.4% of inter‐seasonal notifications, respectively, and typically had the highest number of tests and notifications each inter‐seasonal period. However, the Tropical region had the highest overall inter‐seasonal proportion positive (Figure 1). The Tropical region's proportion positive each inter‐seasonal period ranged from 0.9% (2009/10) to 20.6% (2018/19), whilst the Southern and Central regions had lower and narrower proportion positive ranges (Southern: 0.4%‐9.2%; Central: 0.4%‐10.3%). Plots of weekly proportions positive by region also indicate periods of markedly increased activity within inter‐seasonal periods in the Tropical region. The weekly proportion positive exceeded 20% in the Tropical region in 5/11 inter‐seasonal periods (2010/11, 2011/12, 2013/14, 2014/15, 2018/19). During the 2010/11 period, 778 notifications were reported in the Tropical region (exceeding Central (510) and Southern (517) region notifications) and the overall proportion positive reached 17.4%.

The overall ratio for inter‐seasonal to seasonal periods was 0.19 for notifications and 0.74 for testing. When assessed with other categories, higher testing ratios, comparing inter‐seasonal to seasonal periods, were observed among children younger than 5‐years of age (0.93), males (0.77), and the tropical region (0.94) (Table 1). The proportion positive ratio was also highest for the Tropical region (0.63) as compared to all other regions and demographic groups. Notification count ratios were lowest among children in the 5‐<10‐year age group (0.12) and for influenza B (0.12). Among influenza strains, the notification count ratio was highest for the Yamagata Lineage (0.68). However, counts of subtyped influenza B notifications were low.

### 3.2 Intensity and strain dominance

**Figure 2:**
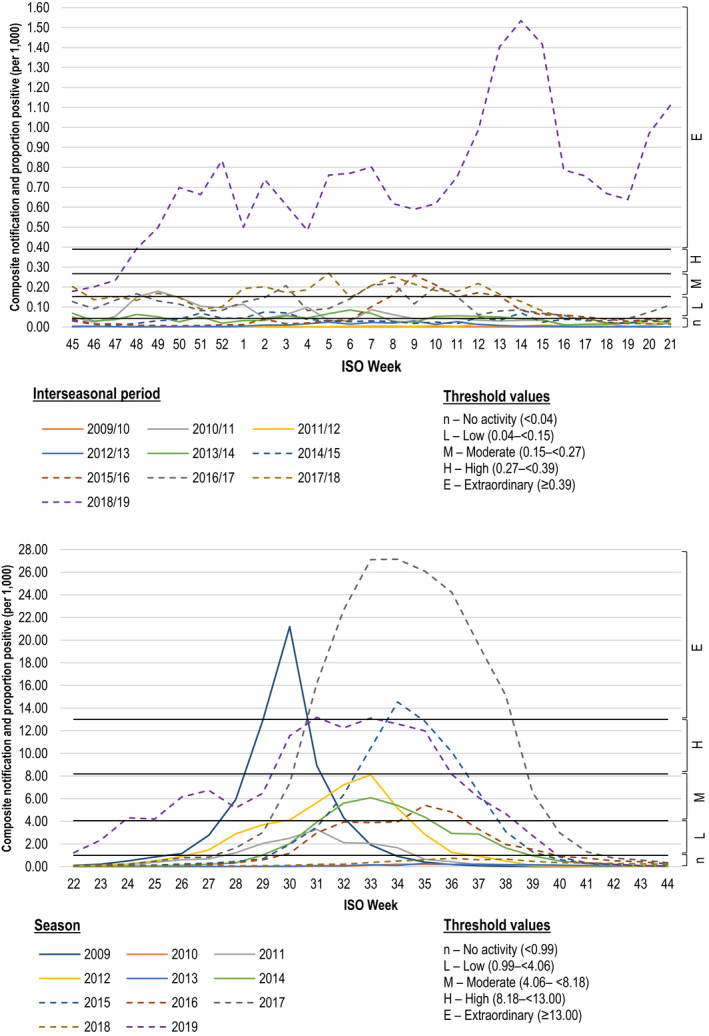
Weekly composite laboratory‐confirmed influenza rate (per 1000 population) and proportion positive and influenza intensity thresholds, Queensland 2009‐2019. (A) Inter‐seasonal periods, 2009/10 to 2018/19. (B) Seasonal periods, 2009 to 2019

Using the intensity threshold levels, the majority of inter‐seasonal periods were categorized as either “no activity” (2009/10, 2011/12, 2012/13) or “moderate” (2010/11, 2015/16, 2016/17) (Figure 2A). Composite values (product of notification rate and proportion positive) during the 2016/17 and 2017/18 periods were above the seasonal threshold for a total of 31 and 40 weeks, respectively. Only the 2018/19 period was classified as “extraordinary”. Three seasons were classified as “no activity” (2010, 2013, 2018), whilst four exceeded the “extraordinary” threshold (2009, 2015, 2017, 2019) (Figure 2B).

**Figure 3:**
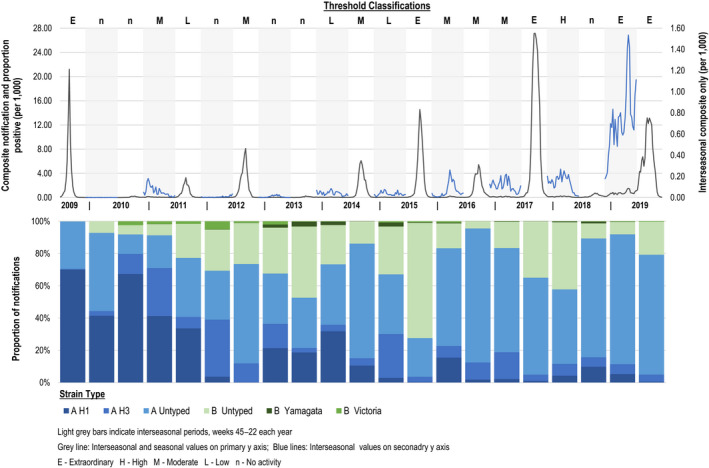
Weekly composite laboratory‐confirmed notification rate and proportion positive, and threshold classification and strain circulation by seasonal and inter‐seasonal periods, Queensland 2009‐2019

Influenza A was the dominant strain across all periods, both inter‐seasonal and seasonal, apart from the 2015 season where 72.4% of notifications were influenza B (Untyped: 98.7%, Victoria: 0.8%, Yamagata: 0.5%) (Figure 3). Overall 25.5% of notifications were influenza B (including 2009 season); 27% of seasonal notifications (including 2009 season), and 18.5% of inter‐seasonal notifications. During the 2017 season and 2017/18 inter‐seasonal period, influenza B accounted for 35% and 42% of notifications, respectively. Co‐circulation of influenza B (≥20% of notifications) occurred in 45% (5/11) of seasons and 50% (5/10) of inter‐seasonal periods. During seasons (excluding the 2009 pandemic) 87% (117 324/134 972) of influenza A notifications were untyped and 91% (43 260/55 354) of influenza B notifications were untyped. Proportions untyped increased to 76% (22 405/29 608) for influenza A and 94.5% (6368/6740) for influenza B during inter‐seasonal periods.

**Figure 4:**
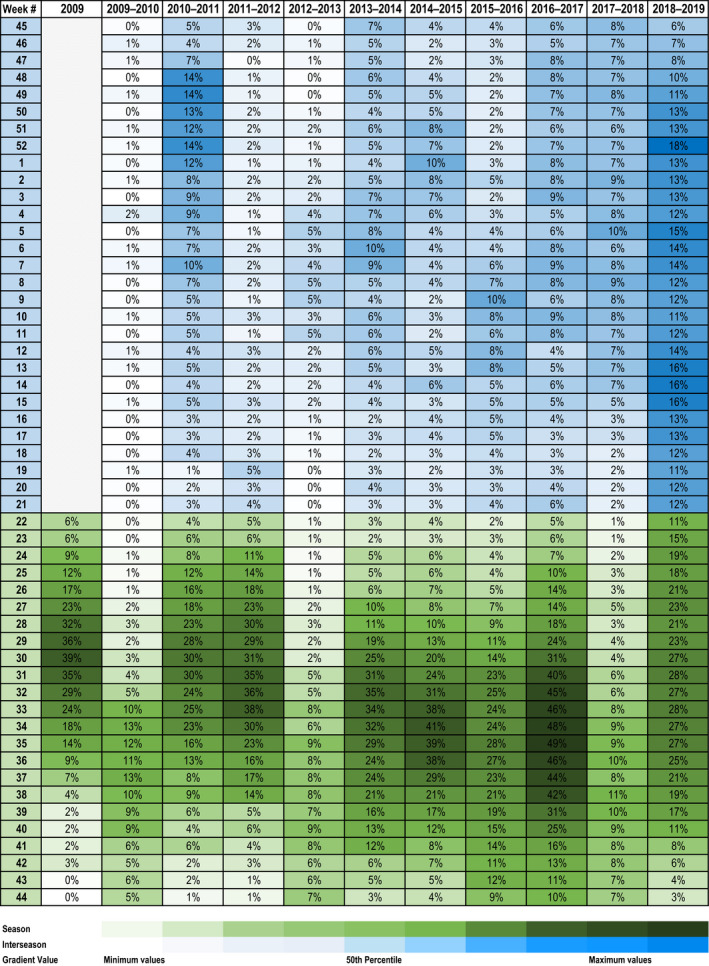
Heat map of weekly proportions of influenza tests positive by seasonal and inter‐seasonal periods, Queensland 2009‐2019

Seasonal activity, as defined by the weekly composite and threshold classifications, following the influenza A dominant “extraordinary” seasons (2009, 2017) was classified as “no activity”, and were followed by high inter‐seasonal activity; the “extraordinary” 2018/19 period and “moderate” 2010/11 period with an elevated proportion positive (Figures 3 and 4). The 2015 influenza B “extraordinary” season was followed by a moderate season (2016) and moderate inter‐seasonal period (2016/17).

## 4 DISCUSSION

By examining Queensland notification and laboratory testing data we found markedly increased inter‐seasonal influenza notifications and testing over the 10‐year period. Gradual increases in weekly inter‐seasonal proportions positive and intensity classifications over time were also apparent and provide evidence to suggest a real increase in inter‐seasonal influenza in Queensland.

In contrast to earlier inter‐seasonal periods where weekly proportions positive exceeding 5% were relatively uncommon, from 2016/17 onwards weekly proportions positive generally exceeded 5%. Similarly, only one of six earlier inter‐seasonal periods were classified above “low” intensity, whilst four consecutive periods between 2015/16 to 2018/19 were classified as “moderate”, “high” or “extraordinary”. The 2017/18 inter‐seasonal period is noteworthy as intensity did not reach the extraordinary levels evident in the 2018/19 period, yet there was sustained intensity with 15 of 29 weeks within the moderate or high intensity range and 17 of 29 weeks with a proportion positive between 7%‐10%. The anticipated baseline of influenza activity during inter‐seasonal periods appears to have shifted to now include sustained moderately intense activity. It is unlikely that increases in weekly proportions positive and intensity classifications are driven by increased notifications alone. Testing and the proportion positive values have also increased over time. Factors such as changing climatic patterns, stepwise increases in vaccine uptake, and increased global interconnectedness and tourist arrival numbers may play a role in increasing inter‐seasonal influenza activity.^7^, ^14^ Indeed, a high proportion of influenza cases among children attending a sentinel hospital in temperate Australia during 2019 were found to be travel related.^9^ Importing diverse viruses from northern hemisphere seasonal epidemics or other tropical regions into Australian summers is highly relevant for Queensland which has four of the top ten Australian tourism regions for international visitors.^3^, ^5^, ^15^, ^16^ A shift of peak seasonal activity into the latter parts of the seasonal period may also affect the distribution of activity in inter‐seasonal periods.

In Queensland it also appeared that seasons with extraordinary levels of influenza transmission intensity, such as the 2009 pandemic and 2017 season, had an impact on the intensity of the subsequent seasons and interseasons. Subsequent seasonal activity is reduced and shifted into the following inter‐seasonal period. Seasons subsequent to those characterized as “extraordinary” in intensity, registered on intensity thresholds as “no activity”. Yet their subsequent inter‐seasonal periods (2010/11, 2018/19) are well established by previous national studies and our findings as having heightened transmission; increased notifications associated with higher proportions positive.^3^, ^7^, ^8^, ^17^ However, this intensity pattern was only evident in “extraordinary” seasons which were influenza A dominant. This effect may be replicated or reinforced by increasing vaccination use in Australia. Increasing coverage with a well‐matched vaccine may mimic immunity levels established by a strong influenza season. This may have the effect of limiting activity in the subsequent season, pushing up intensity in the following inter‐seasonal period. Modelling could be used to explore the impact season size, vaccine match and natural vs vaccine‐induced immunity has on future seasonal and inter‐seasonal intensity.

Consistent with previous literature, we found that the Tropical region contributed considerably to increased inter‐seasonal influenza activity during 2010/11 and 2018/19 periods.^3^, ^7^, ^8^ Additionally, our analysis highlights divergent trends in tropical activity timing and strain circulation. During both periods, we found different weekly peak activity and strain circulation between the tropical, sub‐tropical, and temperate regions of Queensland. For example, during the 2018/19 period, whilst influenza activity the Central and Southern region began to return to a baseline inter‐seasonal level in January 2019, notifications and the proportion positive continued increasing in the Tropical region until a peak in April 2019. Similar discrete trends of activity and strain circulation in the Tropical region was evident throughout the 10‐year period.

The Tropical region appears to have a typically higher level of influenza circulation during inter‐seasonal periods compared to sub‐tropical and temperate regions. Higher notification and test counts in Southern and Central regions were most likely associated with higher population density, whilst the overall higher proportion positive in the Tropical region suggests increased activity. Characterising the Tropical region as having higher influenza circulation during inter‐seasonal periods is consistent with recent Australian literature and research on seasonality more broadly.^2^, ^3^, ^7^, ^8^ Rather than fitting into seasonality models of other tropical regions, the overall seasonality of the Tropical region still appears to align to that of temperate Australian regions. Unlike regions nearer to the equator, the Queensland Tropical region did not have stable circulation levels throughout the year, and bi‐modal peaks were not observed, as would be expected in many South Asian countries.^6^, ^18^, ^19^ Tropical influenza activity, as indicated by notifications and the proportion positive, increased by almost 50% during seasonal periods and at a similar time to the sub‐tropical and temperate regions. As well as this the same relatively heightened circulation and sporadic outbreaks should be anticipated in Tropical regions during inter‐seasonal periods.

Evaluation of the ratio of inter‐seasonal to seasonal tests also indicated that influenza testing remains at relative constant levels regardless of season/inter‐seasonal period for the Tropical region and among children younger than five years of age. This potentially reflects awareness of influenza risk regardless of time point among health practitioners. For the tropical region, health practitioners likely recognize influenza as a cause of respiratory illness during inter‐seasonal periods, whilst for children younger than 5 years of age it is likely related to both clinical presentations of influenza and severity. Respiratory symptoms in children lack pathogen specificity, and along with the availability of multi‐respiratory virus PCR panels in Queensland including the GeneXpert influenza A/B/RSV test, infants and children with even moderate respiratory illness are likely to be tested for a number of pathogens, including influenza.^20^ In this context, influenza seasonality may be less likely to have an impact on a practitioner's decision to test for influenza.

Influenza A was the dominant strain across the 10‐year period, yet there was frequent co‐circulation of influenza B, particularly in inter‐seasonal periods. Overall 18.5% of inter‐seasonal notifications were influenza B and per‐period inter‐seasonal proportions of influenza B ranged up to 42.2% (2017/18). The proportion of inter‐seasonal notifications due to influenza B are rarely presented: this proportion in Okinawa, Japan (2007‐2014) was considerably lower than Queensland values at 4.5%.^21^ Our inter‐seasonal influenza B proportion was most similar to the Australian seasonal average (17.1%) and global seasonal average (22.6%).^22^, ^23^ The majority of influenza B notifications were not characterized, particularly during inter‐seasonal periods where overall less typing was observed. The proportion of seasonal and inter‐seasonal influenza A notifications subtyped also appears to have reduced over the 10‐year period. A similar diminishing trend in influenza A subtyping is nationally evident.^24^, ^25^ Influenza B has been considered to play an important role in inter‐seasonal influenza and summer epidemics both in Australia and globally, particularly related to viral persistence.^3^, ^5^, ^21^, ^23^ A recent Australian analysis found strong evidence for viral persistence in influenza B Victoria Lineage.^3^ Influenza B can represent a large proportion of seasonal and inter‐seasonal period notifications with potentially differential impacts of severity, timing and demographic distribution. Therefore, greater resources for typing, particularly during inter‐seasonal periods, are required.

Our study has several limitations. Laboratory‐confirmed influenza notifications and testing data have limited representativeness as many community influenza cases are not laboratory confirmed and data are subject to presentation and testing biases. The study was also restricted to the use of public laboratory data and therefore may not be fully representative of Queensland testing activity, with gaps most likely in capturing community‐managed illness. As such, our understanding of overall influenza activity involved a combination of testing, notifications and proportion positive. Improvements to PCR testing sensitivity over the study period may have occurred and could not be controlled for in our analyses. Intensity thresholds have also not been previously calculated for inter‐seasonal periods.

Inter‐seasonal influenza represents a complex period of influenza activity and transmission, which may result in a considerable burden of disease in tropical and other climates. By understanding the epidemiological trends of influenza over climatic regions over time a clearer understanding of the interplay between testing, activity and intensity, and strain circulation can be achieved. In particular, our study illustrates that the Tropical region of Queensland has a higher baseline of activity during inter‐seasonal periods with periods of divergent activity and strain circulation as compared to temperate regions. Inter‐seasonal periods have recently gained attention among Australian influenza researchers, yet there are reported increases in inter‐seasonal or summer influenza across a number of countries.^14^, ^21^, ^26^, ^27^, ^28^, ^29^ This study gives further weight to recommendations for year‐round influenza surveillance to better understand its epidemiology during this infrequently studied time period. Whilst the largest burden of inter‐seasonal influenza is in tropical regions, heightened inter‐seasonal influenza activity occurs across climatic zones, and as demonstrated by the 2018/19 transmission intensity, activity can reach extraordinary levels. Collection and analysis of high‐quality surveillance data should not be limited to seasonal periods. Furthermore, strain circulation, timing and transmission intensity varies between climatic regions, and even among tropical regions, seasonal trends vary. Therefore, countries and regions, specifically those with tropical or varied climates, should look to undertake routine year‐round influenza surveillance stratified to account for climatic zones and strain circulation. With potential interconnectedness between seasonal and inter‐seasonal influenza activity, surveillance of inter‐seasonal influenza may also have value in contextualizing or anticipating seasonality and intensity over time. Further research into the potential flow on impacts of seasonal intensity and strain circulation on inter‐seasonal and seasonal periods over a longer timeframe may provide greater insights into drivers of influenza seasonality.

## CONFLICT OF INTEREST

None declared.

## AUTHOR CONTRIBUTIONS

**Elenor J. Kerr:** Conceptualization (lead); formal analysis (lead); methodology (lead); visualization (lead); writing‐original draft (lead); writing‐review & editing (lead). **Jonathan Malo:** Conceptualization (supporting); methodology (supporting); supervision (equal); visualization (supporting); writing‐review & editing (equal). **Kaitlyn Vette:** Methodology (supporting); visualization (supporting); writing‐review & editing (equal). **Graeme R. Nimmo:** Conceptualization (supporting); resources (supporting); writing‐review & editing (equal). **Stephen B. Lambert:** Conceptualization (supporting); methodology (supporting); supervision (equal); visualization (supporting); writing‐review & editing (equal).

## Funding information

Elenor Kerr is supported by an ASEAN‐Australia Master of Philosophy (Applied Epidemiology) scholarship funded by the Department of Foreign Affairs and Trade.

## ETHICAL APPROVAL

Approval was granted by the Australian National University Human Research Ethics Committee (Protocol 2019/442) and approval for access to Queensland Health information was granted under section 284 of the Queensland Public Health Act 2005.

## PATIENT CONSENT STATEMENT

All data used by this study was from state‐wide routine surveillance of a notifiable condition and were de‐identified.

## PERMISSION TO REPRODUCE MATERIAL FROM OTHER SOURCES

No permissions required.

### PEER REVIEW

The peer review history for this article is available at https://publons.com/publon/10.1111/irv.12828.

## Data Availability

Data are subject to third part restrictions. The data that support the findings of this study are available from Queensland Health. Restrictions apply to the availability of these data, which were granted for use under section 284 of the Queensland Public Health Act 2005. Data are available from authors with the permission of Queensland Health.

## References

[1] Moa AM , Adam DC , MacIntyre CR . Inter‐seasonality of influenza in Australia. Influenza Other Respi Viruses. 2019;13(5):459–464. 10.1111/irv.12642 PMC6692536 30929310

[2] Tamerius JD , Shaman J , Alonso WJ , et al. Environmental predictors of seasonal influenza epidemics across temperate and tropical climates. PLoS Pathog. 2013;9(3):e1003194. 23505366 10.1371/journal.ppat.1003194 PMC3591336

[3] Patterson Ross Z , Komadina N , Deng YM , et al. Inter‐seasonal influenza is characterized by extended virus transmission and persistence. PLoS Pathog. 2015;11(6):e1004991. 26107631 10.1371/journal.ppat.1004991 PMC4479464

[4] Nelson MI , Simonsen L , Viboud C , et al. Stochastic processes are key determinants of short‐term evolution in influenza A. PLoS Pathog. 2006;2:e125. 17140286 10.1371/journal.ppat.0020125 PMC1665651

[5] Rambaut A , Pybus OG , Nelson MI , Viboud C , Taubenberger JK , Holmes EC . The genomic and epidemiological dynamics of human influenza A virus. Nature. 2008;453:615‐659. 18418375 10.1038/nature06945 PMC2441973

[6] Hirve S , Newman LP , Paget J , et al. Influenza seasonality in the tropics and subtropics ‐ when to vaccinate? PLoS One. 2016;11(4):e0153003. 27119988 10.1371/journal.pone.0153003 PMC4847850

[7] Barr IG , Deng YM , Grau ML , et al. Intense interseasonal influenza outbreaks, Australia, 2018/19. Euro Surveill. 2019;24(33):1900421. 10.2807/1560-7917.ES.2019.24.33.1900421 PMC6702793 31431210

[8] Kelly HA , Grant KA , Tay EL , Franklin L , Hurt AC . The significance of increased influenza notifications during spring and summer of 2010–11 in Australia. Influenza Other Respi Viruses. 2013;7(6):1136‐1141. 10.1111/irv.12057 PMC4634258 23176174

[9] Deng L , Mazzocato P , Saravanos G , Leder K , Britton PN . A high proportion of interseasonal childhood infuenza cases in 2019 were travel related. Public Health Res Pract. 2020;30(2):e3022012. 10.17061/phrp3022012 32601658

[10] Appuhamy RD , Beard FH , Phung HN , Selevy CE , Birrell FA , Culleton TH . The changing phases of pandemic (H1N1) 2009 in Queensland: an overview of public health actions and epidemiology. MJA. 2010;192(2):94‐97. 20078411 10.5694/j.1326-5377.2010.tb03427.x

[11] World Health Organization . Pandemic Influenza Severity Assessment (PISA). World Health Organization. https://apps.who.int/iris/bitstream/handle/10665/259392/WHO‐WHE‐IHM‐GIP‐2017.2‐eng.pdf;jsessionid=7AB3501D1EBACF0CFE28FC8942493A26?sequence=1. Accessed July 24, 2019

[12] Tay EL , Grant K , Kirk M , Mounts A , Kelly H . Exploring a proposed WHO method to determine thresholds for seasonal influenza surveillance. PLoS One. 2013;8(10):e77244. 24146973 10.1371/journal.pone.0077244 PMC3795663

[13] Goldstein E , Viboud C , Charu V , Lipsitch M . Improving the estimation of influenza‐related mortality over a seasonal baseline. Epidemiology. 2012;23(6):829‐838. 22992574 10.1097/EDE.0b013e31826c2dda PMC3516362

[14] Towers S , Chowell G , Hameed R , et al. Climate change and influenza: the likelihood of early and severe influenza seasons following warmer than average winters. PLoS Curr. 2013;5:1‐8. 10.1371/currents.flu.3679b56a3a5313dc7c043fb944c6f138 PMC3770759 24045424

[15] Uyeki TM , Zane SB , Bodnar UR , et al. Large summertime influenza A outbreak among tourists in Alaska and the Yukon Territory. Clin Infect Dis. 2003;36(9):1095‐1102. 12715302 10.1086/374053

[16] Tourism Research Australia . International Visitor Survey: YE. December 2019. Austrade. https://www.tra.gov.au/ArticleDocuments/185/IVS_TOURISM_RESULTS_YE_DEC_2019.xlsx.aspx. Accessed April 17, 2020

[17] Moa A , Trent M , Menzies R . Severity of the 2019 influenza season in Australia‐ a comparison between 2017 and 2019 H3N2 influenza seasons. Glob Biosecurity. 2019;1(3):1‐6.

[18] Caini S , Andrade W , Badur S , et al. Temporal patterns of influenza A and B in tropical and temperate countries: what are the lessons for influenza vaccination? PLoS One. 2016;11(3):e0152310. 27031105 10.1371/journal.pone.0152310 PMC4816507

[19] El Guerche‐Seblain C , Caini S , Paget J , Vanhems P , Schellevis F . Epidemiology and timing of seasonal influenza epidemics in the Asia‐Pacific region, 2010–2017: implications for influenza vaccination programs. BMC Public Health. 2019;19(311):1‐10. 30898100 10.1186/s12889-019-6647-y PMC6429768

[20] Kaczmarek MC , Schlebusch S , Ware RS , Coulthard MG , McEniery JA , Lambert SB . Diagnostic testing in influenza and pertussis‐related paediatric intensive care unit admissions, Queensland, Australia, 1997–2013. Comm Dis Intell. 2017;41(4):E308‐E317. 10.33321/cdi.2017.41.41 29864384

[21] Sunagawa S , Iha Y , Taira K , et al. An epidemiological analysis of summer influenza epidemics in Okinawa. Intern Med. 2016;55(24):3579‐3584. 27980256 10.2169/internalmedicine.55.7107 PMC5283956

[22] Caini S , Huang QS , Ciblak MA , et al. Epidemiological and virological characteristics of influenza B: results of the Global Influenza B Study. Influenza Other Respi Viruses. 2015;9(Suppl 1):3‐12. 10.1111/irv.12319 PMC4549097 26256290

[23] Moa AM , Muscatello DJ , Turner RM , MacIntyre CR . Epidemiology of influenza B in Australia: 2001–2014 influenza seasons. Influenza Other Respi Viruses. 2017;11(2):102‐109. 10.1111/irv.12432 PMC5304570 27650482

[24] Department of Health . Influenza (Laboratory‐confirmed) National Notifiable Disease Surveillance Notifications in Australia 2008–2017. Australian Government Department of Health. http://www9.health.gov.au/cda/source/pub_influ.cfm. Accessed April 17, 2020

[25] WHO Collaborating Centre for Reference and Research on Influenza (VIDRL) . 2018 Annual Report. WHO Collaborating Centres for Reference and Research on Influenza. http://www.influenzacentre.org/documents/publications_reports/Annual%20Report%202018.pdf. Accessed April 17, 2020

[26] Wolf DG , David R , Eitan K , et al. A summer outbreak of influenza A virus infection among young children. Clin Infect Dis. 2004;39(4):595‐597. 15356830 10.1086/422457

[27] Gurav YK , Chadha MS , Tandale BV , et al. Influenza A(H1N1)pdm09 outbreak detected in inter‐seasonal months during the surveillance of influenza‐like illness in Pune, India, 2012–2015. Epidemiol Infect. 2017;145(9):1898‐1909. 28367767 10.1017/S0950268817000553 PMC9203343

[28] Ghedin E , Wentworth DE , Halpin RA , et al. Unseasonal transmission of H3N2 influenza A virus during the swine‐origin H1N1 pandemic. J Virol. 2010;84(11):5715‐5718. 20237080 10.1128/JVI.00018-10 PMC2876599

[29] Tsou TP , Su CP , Huang WT , Yang JR , Liu MT . Influenza A(H3N2) virus variants and patient characteristics during a summer influenza epidemic in Taiwan, 2017. Euro Surveill. 2017;22(50):17‐00767. 10.2807/1560-7917.ES.2017.22.50.17-00767 PMC5743095 29258649

